# Intersectional discrimination and mental health inequalities: a qualitative study of young women’s experiences in Scotland

**DOI:** 10.1186/s12939-024-02133-3

**Published:** 2024-02-29

**Authors:** Laura Tinner, Ana Alonso Curbelo

**Affiliations:** 1 Population Health Sciences, Bristol Medical School, University of Bristol, Bristol, UK; 2 School of Social and Political Sciences, University of Glasgow, Glasgow, UK

## Abstract

**Background:**

In 2021, Scotland became the first UK country to launch a Women’s Health Plan. This policy signals increasing commitment to broader ambitions surrounding gender equality in health. Research shows a connection between discrimination and health, representing a contributor to health inequalities. There remains sparse evidence on how certain groups experience discrimination that could be useful for policymaking. This research set out to address this evidence gap through exploring how discrimination shapes young women’s experiences of mental health and inequalities in Scotland.

**Methods:**

We interviewed women aged 16-25 years (*n*=28), living in Scotland, UK, adopting an intersectional approach to recruitment and data analysis. We used a semi-structured topic guide to facilitate open discussion about discrimination and health. Transcripts were analysed by two researchers using Thematic Analysis and NVivo software.

**Findings:**

We identified three themes that illuminate intersectional discrimination and the impact on mental health. The first outlines how experiences of discrimination in school, work and public spaces (and the anticipation of such) creates stress leading to mental health problems, particularly for participants from ethnic minority groups. The second highlights the lack of support for mental health, both at structural and interpersonal levels, which was viewed by young women as a form of intersectional discrimination, largely because of their gender and age. Finally, we developed a mid-level theory termed the ‘chain of dismissal’ that displays that for both physical or mental health symptoms, young women’s concerns are immediately “written off” as anxiety-related and in turn a natural attribute of young women. These themes show that discrimination has the potential to amplify mental health problems for young women and is a likely contributor to health inequalities.

**Conclusions:**

Structural disadvantages such as racism intersect with gender and age to compound the experience of discrimination for marginalised young women. To improve mental health and reduce health inequalities for young women, multi-level approaches are needed, with strong consideration of how the structural and cultural landscape as well as assumptions made by healthcare professionals have critical implications for young women’s health.

**Supplementary Information:**

The online version contains supplementary material available at 10.1186/s12939-024-02133-3.

## Background

In 2021, Scotland became the first UK country to launch a Women’s Health Plan [1]. This policy signals increasing commitment to broader ambitions surrounding gender equality in health as articulated by the United Nations (UN) Sustainable Development Goal 5. We know that women have unique health issues and there are several conditions (e.g., hypertension) that both women and men experience, but can present in women differently, leading to longer diagnosis times and deleterious outcomes [2]. Research also highlights gender inequalities in terms of healthcare, with women tending to experience greater challenges in access as well as lower standards of healthcare [3]. There are not only differences between men and women, but also among women – they are not a homogenous group [4]. For example, there is vast evidence of socioeconomic [5] and racial inequalities [6] in health in the UK across a number of health outcomes, which many women are likely to experience. Yet there is still a lack of evidence on how certain groups experience health inequalities and, despite advancing efforts, Scotland still experiences some of the largest health inequalities in Europe [7]. This research set out to address this evidence gap through exploring how discrimination shapes different mental health inequalities among young women in Scotland.

### Mental health inequalities and young women

Mental health is a major public health priority for Scotland [8]. There is widespread evidence demonstrating inequalities in mental health outcomes such as anxiety and depression [9, 10], yet the pathways are complex and not well-understood. Young people are a key population in this field, given a growing concern over the rise in mental health conditions among adolescents [11] and the fact that improving health at earlier life stages could have far-reaching effects into adulthood [12].

Recent data shows notable gender differences in mental health inequalities in young people [13]. Evidence from the Department of Health and Social Care in 2022 [14] found that young women aged 17-24 were nearly three times more likely to have a mental health disorder than young men, which represented a gender difference not present in younger adolescents aged 11-16 years, where there was little different in the rates of probable mental health disorders between girls and boys [14]. The Covid-19 pandemic has exacerbated these mental health pressures for young people [11], especially for young women [15]. Further, the recent Scottish Government’s Health and Wellbeing Census (2021-2022) highlighted evidence that girls report poorer mental and physical health than boys in Scotland [16]. In this survey, across a range of wellbeing and mental health measures, girls reported less-positive perceptions than boys their age. This Scottish data aligns with international research that suggests that girls are more likely to display symptoms of mental health conditions such as depression and anxiety [17]. Despite these data, researchers such as Jordan and Chandler [18] highlight the gendered framing of mental health in that suicide has been discursively framed as a male issue, which can position young women and girls as in less need of support and treatment as their experiences of mental health problems. Further, Mizock and Brubaker [19] found that women often had the legitimacy of their symptoms questioned by mental health providers, highlighting gendered inequalities in healthcare access as well as outcomes.

There is evidence from the UK [20, 21] and the USA [22, 23] that young people from ethnic minority backgrounds and those experiencing poverty have increased likelihood of experiencing mental health difficulties. There is therefore rationale to approach our study from the understanding that many young women may be experiencing several different inequalities at one time. To date, these inequalities have largely been examined a single-entities and rarely understood as mutually constituted and intersecting experiences [10]. It is of specific relevance to this paper that we analyse young women’s experiences through a lens that enables us to capture various forms of mental health inequalities. We do this through a focus on discrimination and by using intersectionality to frame our understanding.

### Discrimination and mental health

One fundamental cause of inequalities in mental health is discrimination [24]. Discrimination refers to the unfair treatment of individuals or groups based purely on membership to a subordinated social group, arising from struggles of power and privilege [25]. The experience of discrimination related to social identities including gender, age, socioeconomic background, race/ethnicity, disability and sexual orientation can impact on health outcomes, healthcare access and lead to social exclusion and isolation [26]. Experiences of discrimination are often categorised to three ‘levels’: macro, meso and micro [27]. The macro level, also referred to as the structural level, conveys how ‘policies, practices, norms, power structures, and dynamics restrict, undermine, subjugate, or oppress marginalised groups and individuals’ ([27], p.3). The meso or interpersonal level refers to interactions with individuals that give way to discrimination and differential treatment based on ‘prevalent negative stereotypes in the society’ ([27], p.3). Thirdly, the micro level, which is also called the individual level, refers to the ‘experiences from macro and meso levels give rise to negative internalised feelings among those affected’ ([27–29], p.3,).

Mental health and discrimination have broadly been researched in relation to the impact of ‘perceived discrimination’ on mental and physical health outcomes [30, 31]. There is considerably less work on young women and girls’ appraisals of gender discrimination and the effects on their wellbeing or mental health, and even less on how age discrimination is present in these experiences. This is surprising given that adolescence leads to psychological developments that render discrimination more discernible and personally relevant [32]. Discriminatory experiences related to one’s gender are possible contributors to psychological or emotional challenges in girls [32]. However, it is not just gender or age discrimination (or indeed any one form of discrimination), that is likely to have impacts on young people’s mental health [33]. Discrimination therefore can potentially meaningfully impact on the lives of young women experiencing multiple disadvantage [33, 34]. There is thus a strong rationale for investigating how young women experience discrimination and the role it plays in their health and inequalities using intersectionality as a framework.

### Intersectionality

We adopted intersectionality as a theoretical framework to inform our sampling, recruitment and analysis of young women’s experiences of discrimination. Intersectionality, coined by Kimberlé Crenshaw [35], underscores that there are many different characteristics of a person that make up their identity. It was borne out of Black women’s experiences of simultaneous racism and sexism [36, 37]. However, its use has expanded to include other identities, and it has been particularly influential in drawing attention to how other identity facets such as ethnicity, social class, disability and sexuality might traverse gender lines and shape individuals’ unique lived experiences [38]. There is building evidence surrounding mental health and intersectional discrimination in young people, much of which has been conducted in North America. For instance, a critical review found that racism and heterosexism predicted symptoms of depression and that those in positions of multiple disadvantage exhibit high risk for mental health problems [39]. Further, Mallory and Russell [40] found that intersectional minority stressors undermine the mental health of sexual minority youth of colour.

Intersectionality is especially important for women’s health, as Varcoe et al ([41] p.12) note, *‘*inherent in the concept of women’s health is the paradoxical challenge that differences among women are often greater than the differences between women and their implied binary opposite, men’. Understanding the realities of these intersections and what they mean for health inequalities is an essential step in building effective solutions.

### Research aims

Given the current gaps in evidence, this research aimed to use intersectionality as a framework to: (1) examine how young women’s experience of discrimination within and outside of the healthcare system, and (2) understand in what ways these discriminatory experiences shape health, inequalities and access to treatment. Our goal was for this work to be informative for future phases of the Women’s Health Plan and policy to reduce inequalities for young women in Scotland.

## Materials and Methods

### Research Design

A qualitative study was designed as part of a package of research to contribute to one of the actions in the Women’s Health Plan [1]:

- Build an evidence base on women’s health inequalities, with specific focus on the impact of sexism, racism, ableism, and other forms of discrimination including homophobia and transphobia on women’s health.

The research was undertaken by academic researchers who were selected for policy fellowship placements within the Scottish Government. The work, therefore, remained close to policy priorities but the visiting researchers had autonomy over the research design and data analysis process. We chose one-to-one interviews for data collection, following a series of preliminary focus groups with a broader age range of women. The research was designed with a broader remit of ‘women’s health’, which was reflected in the research questions in that they did not have a strong mental health focus. Despite this, mental health dominated many of the discussions with young women and warranted specific attention in this article.

### Participant recruitment

Recruitment was supported by a stakeholder group, The ‘Health and Social Care Alliance Scotland’, referred to as the ALLIANCE throughout this paper. We made contact with Scottish charities, schools, colleges, universities and on social media to advertise the project. Participants were eligible if they identified as a woman or they were eligible to engage with women’s health services; they were aged 16-25 years and currently resided in Scotland. People who identified as trans or non-binary were eligible for recruitment. None of our sample participants identified as trans or non-binary, but one participant said they were questioning their gender identity. Interested participants emailed the research team and were sent a participant information sheet, privacy notice, consent form and demographic characteristic survey. The survey questions were designed to capture the diversity within the sample and allow the recruitment strategy to be altered if there were obvious gaps. Survey questions were modelled on standardised Scottish Government Collecting Equality Data advice (2022) and the Scottish Census (2022).

### Data collection

Interviews took place between October 2022 and March 2023 either via video conferencing (*n*=25) or telephone (*n*=3). We developed a semi-structured topic guide to follow throughout the interviews. All participants signed an informed consent form before the interviews, having read through the participant information sheet and privacy notice and had the opportunity to ask questions.

The topic guide, which focused on young women’s experiences of discrimination and how these relate to health, was reviewed by an advisory group made up of research and policy personnel working on Scotland’s Women’s Health Plan. Our topic guide (Appendix 1) was developed through conversations with members of a lived experience group, wider stakeholders and literature on adolescence and young adults, discrimination, intersectionality and women’s health. It was kept intentionally broad so that data were driven by the area of health and discrimination each participant saw as most important. The topic guide was piloted with a young woman in England who was not eligible for the study. As a result of the pilot, we made some changes to the framing of certain questions to illicit more narrative responses and to enable participants to think of the intersectional nature of experiences.

We began each interview by reading the points on the consent form and summarising the overall topic. We reminded participants they could withdraw at any stage. We worked through the topic guide and used probing questions based on participant answers. All interviewees were sent a thank you voucher via email along with a standard signposting support contact sheet.

### Data analysis

Transcripts were analysed by [LT] and [AAC], who are both White women, under the age of 35, with university education, residing in the UK and one from another European country. We found that being women undoubtedly added a sense of relatability within the interviews. We often heard examples of discrimination that we had experienced ourselves. Conversely, there were several experiences, particularly related to racism, that we could not personally speak to. We had multiple discussions about our own position and aimed to stay as grounded within the data as possible, drawing out experiences both similar and contrasting to our own. However, our positions will have impacted how the interviewees interacted with us, the examples they gave and the themes we subsequently drew out.

We used reflexive Thematic Analysis, guided by Braun and Clarke [42] and used NVivo 12 [43] to manage the data during the analysis. We kept an Excel sheet of reflexive and substantive notes related to each interview. We began the iterative coding process when we had five transcripts, familiarising ourselves with the data alongside our notes. We then independently open coded each transcript. This was followed by axial coding in which we revisited the transcripts and grouped together similar codes, eventually leading to theme generation. It became clear during this process that ‘mental health’ warranted separate in-depth investigation, given the richness of data on this topic and that every participant mentioned it. We decided to analyse the data for mental health themes and report in this present paper. There is an additional publication that presents the other part of our analysis, which expands on themes generated from the data that were not directly mental health-related (e.g., contraception and reproductive health, ageist sexism).

The two researchers met regularly and discussed the codes and themes. These meetings were used to practice reflexivity, to identify patterns, and discuss conflicts, complexity, paradoxes and our differing interpretations [44]. Participants are labelled with a number (e.g., P1).

### Ethical considerations

Ethical approval for this study was granted by the Scottish Government Health and Social Care Analysis Division, where the researchers were based as part of an embedded researcher fellowship. Confidentiality and anonymity were ensured by replacing names with interview codes and removing potentially identifying information from the transcripts. Data were stored securely on an electronic server, with access restricted to the researchers on the team.

## Results

### Respondent characteristics

Table 1 contains the characteristics of the participants.

**Table 1:** Participant demographic Data

	*N*	%
**Gender**		
Female	27	96%
Other	1	0.4%
**Age**		
16-19	14	50%
20-25	14	50%
**Race/ethnicity**		
White	16	57%
Indian	1	4%
Pakistani	3	11%
Arab	5	18%
African	1	4%
Mixed Race	2	7%
**Sexual orientation**		
Straight/heterosexual	19	68%
Bisexual	9	32%
**Religion**		
Islam	6	21%
Church of Scotland	1	4%
Catholicism	4	14%
Sikhism	1	4%
Other Christianity	3	11%
None	12	43%
**Scottish Index of Multiple Deprivation quintiles**		
Quintile 1 (most deprived)	8	29%
Quintile 2	7	25%
Quintile 3	6	21%
Quintile 4	4	14%
Quintile 5 (least deprived)	5	18%
Not found	2	7%
**Employment status**		
Employee or self-employed	18	64%
Unemployed/long term sick	2	7%
Studying	8	28%

### Theme 1. Discrimination resulting in mental health problems

Almost every participant described and exemplified ways that discrimination had a negative impact on their mental health or wellbeing. The data in this theme can be broadly understood as falling within two categories: (1) some participants highlighted a dominant factor by which they felt discrimination was driving mental health issues (e.g., their gender) or (2) some participants, the distinct intersectional nature of the discrimination was explored (e.g., being a woman from an ethnic minority background and/or young). We address examples from each of these types as they contribute to our question around young women’s mental health inequalities in that in several cases, discrimination is intersectional, but in others, there is a dominant experience (e.g., sexism). For many participants, discrimination (at various levels and across settings) led to increased stress levels or anxiety symptoms, affecting their relationships and their sense of self. Gender discrimination was frequently described independently of other forms of discrimination (perhaps unsurprisingly given the scope of the work and the intended sample), with educational and employment settings being common sites for this type of discrimination. The mental health impact of gender discrimination was highlighted by participants working or studying in STEM (Science, Technology, Engineering and Maths) fields [e.g., P23, P28 and P10]:

...It wore me down so much in my last job. It felt just like a constant battle. It was like a second job to worry about my main job. Not good… it definitely affected my stress levels and my mental health really badly. Yes, towards the end of that job, my anxiety was really, really out of control [P23].

Participant 4 revealed a more deeply intersectional experience of discrimination through describing how her age, gender and socioeconomic circumstances shaped her experience of education and created stress and anxiety symptoms. She felt that there were greater expectations at school upon herself and her female classmates than on boys to perform academically and be well-behaved. This gendered social norm created a sense of pressure to constantly prove herself, which has had negative consequences on her mental wellbeing, manifesting in feelings of inadequacy and stress:

It makes us feel like we need to work harder [than boys], even if we don’t need to catch up because we’re at the same level. We still feel like we’re not doing good enough. Which begins to get really stressful as in you’re constantly, constantly trying to impress all your teachers and it’s a lot of work [P4].

For Participant 4, age and socioeconomic position served as additional intersections that compounded this discrimination. She highlighted how she had to work in a fast-food establishment after school and at weekends to assist her family financially, but because of her young age (age 16) she did not get paid as much as her colleagues at 18 and older due to minimum wage laws in the UK. She also highlighted how her position as a teenage woman with challenging socioeconomic circumstances (e.g., being from a single parent household with frequent worries about money) produced gendered and class-based expectations around helping out at home and getting a job – leaving her less time to do school work and further reinforcing the pressure she felt from teachers. This experience is distinctly intersectional given the complex interplay between these types of macro/structural (e.g., age wage laws, socioeconomic conditions) meso/interpersonal (e.g., treatment by teacher) discrimination, based on gendered norms and expectations around education, work and providing help around the home. The positionality of this adolescent women at a relatively disadvantaged socioeconomic position constrained educational opportunities and in turn exacerbated stress levels resulting in mental health issues.

A subset of participants spoke about the racism they faced and how this was a predominant factor that linked discrimination to their mental health, particularly in educational and public settings. Their experiences were almost always intersectional, namely with characteristics such as: nationality, migrancy, language and religion, but also with gender. Young women in our sample from ethnic minority backgrounds described how their discriminatory experiences were magnified due to their intersectional position that created a specific stereotype around their identity. It was often a challenge to decipher for what reason they were being discriminated against:

My teacher [in college] was a bit racist… like maybe because I’m from a different country or maybe because I’m Arab I don’t know… but she was treating me really bad. And you don’t eat and you’re over-thinking [P6]

If you were a girl, and you’re a hijabi girl, you will definitely get bullied, especially if you have no English. Some boys are taking a different image of the hijabi girls. They think she can’t do anything, she can’t communicate with anybody, she needs to stay by herself alone, away from each other [P8]

For these participants, the impact of discrimination on their mental health was largely expressed through descriptions such as “*not eating*” [P8], “*staying off school*” [P7], “*over-thinking*”, [P6] “*not very good mentally*” [P15] as an alternative to more diagnostic descriptions including “*anxiety*” and “*depression*”, which were terms more commonly used by White participants. This distinction could be interpreted as an avoidance by already marginalised young women to label themselves with a mental health status that is potentially stigmatising. This is something we noticed but we unable to explore in depth, as there could be many other reasons for these language choices such as participants not having the words to express how they feel, rather than active avoidance.

Aside from explicit instances of discrimination like those described here, the *anticipation* of discrimination also appeared to create a strain on young women with the potential for worsening mental health. Participants from ethnic minority backgrounds talked about the exhaustion of anticipating racism over and above other forms of discrimination. The cultural and media landscape that produces negative racial stereotypes, as well as experiences of family members, often informed this anticipation and enhanced stress when entering certain settings, even for young women who said they had never experienced interpersonal racism themselves. This expectation of racism regularly made young women feel that they should alter parts of themselves (e.g., name [P10], accent [P15]), career choices [P6] or stay away from certain spaces [P15, P14]) to avoid discrimination, highlighting the micro-level internalisation of broad oppressive systems. These expectations of negative treatment were intersectional and were magnified for young women whose nationality, religion, migrancy and language was also marginalised:

...I am pretty worried about my future, as want to become a nurse and I’d be working in a hospital or GP and I’ll have to wear a scarf and all the people in the hospital will see you [P6].

I introduce myself with a British name, which makes me sad, because I can’t use the name I was given at birth, because people will find it difficult to say. I have to change a part of me in order to not to be discriminated [against] [P10]

While the overriding anticipation here was related to racism, we interpreted the participants worries (e.g., wearing a headscarf) and social practices to mitigate against situations (e.g., changing oneself and avoiding places) as gendered in that they were often adopting them to be “*taken seriously*” [P10], while men from ethnic minority backgrounds may have different concerns and social practices around anticipating discrimination. The consequences of explicit instances of different forms of discrimination and the anticipation of such had detrimental effects on these young women’s mental health. These data show that in some cases there is an overriding force that drives discrimination (e.g., sexism), while in others, intersectionality frames the discriminatory experience and both aggravate mental health issues and are potential contributors to inequalities.

### Theme 2. Lack of mental health support as form of discrimination

Theme 2 draws upon the experiences of almost half of our sample (*n*=13) who either had a diagnosed mental health condition or experienced mental health symptoms. These participants talked about the formal process of trying to get support or treatment for their mental health issues as a young woman. The lack of support for mental health, both at structural and interpersonal levels, was viewed by young women as a form of intersectional discrimination, primarily due to their age and gender. Most of the data in this theme relates to White women, many of whom highlighted their own privileges in their experience. One participant who talked about additional intersecting characteristics (e.g., ethnic minority status, language) provides insight into how unequal access to mental health support is distinctly intersectional. The initial point of access for mental health support, for our participants, was either student support services at schools or universities, or going to the GP. Young women found that the embedded power structures and social norms within these institutions, coupled with real financial and capacity pressures, produced discriminatory practices that resulted in reduced mental health support for young women:

I’ve really struggled with mental health before and not had support like boys the same age as me would have gotten for, like… Even just being stressed out, kind of, they’ll have a lot of support from pupil support teachers and the school in general [P4]

...I sometimes saw that [at school] boys would be disruptive, and they went to pupil support, and stuff. And whereas the girls in my class were usually told that they were hormonal [P17]

If you mention it to [university], “Oh, I’ve had a rough couple of days, I’ve been really depressed, I need an extension” they go, “Och, you’re fine.” But I have pals who are guys who couldn’t hand stuff [assignments] in and they have been able to use that as evidence. One of my [male] pals was referred on [when they told the university] [P22].

Here young women claim there is an understanding, embedded within the educational settings they attend, that young men experience more serious mental health issues than young women and they need more immediate support. This gendered understanding was coupled with systemic issues and long waiting lists within Child and Adolescent Mental Health Services (CAMHS) in the UK that creates barriers for young people in seeking mental health support. While it was acknowledged that individuals of all ages and genders will experience the negative impact of the pressures on the health system, the particular positionality of young women made it “*easier*” [P1] to discourage them from seeking mental health support. Some young women felt trapped by the embedded societal and medical conceptualisations that they (because of their age and gender) are more likely to be experiencing the effects of “*hormones*” [P16] or the “*menstrual cycle*” [P4] than mental health issues. They also felt that young women were viewed as being more capable of “*getting on with it*” [P10] than their male peers– leading many to theorise that being either older or male would alleviate some of these challenges. These societal discourses around gender and mental health were expressed as a distinct form of macro-discrimination, which in turn limited access to resources for this group within the context of extreme pressures on the health system.

These broader social norms permeated interpersonal interactions young women had with healthcare and educational professionals. Discriminatory actions were intersectional and shaped by assumptions about participants’ identity characteristics (namely their age and gender) and served to restrict young women further from accessing health resources:

When I was first having my symptoms of depression, when I was talking to, like, my mum about it, or teachers, they were always just say, “Oh, you’re just, like, a teenage girl, like, you’re just, like, emotional, and very hormonal, because you’re going through puberty, and it’s not actually anything, really.” And then here, five years later, it definitely is not that [P17]

I went back to my GP, but this time it was about anxiety... They were like, “Is it just that she’s on her period?” [P4]

These excerpts point to the specific intersectional aspect of these discriminatory experiences. Referring to participants as a ‘*teenage girl*’ reveals how this particular positionality is framed by healthcare professionals as baring direct relevance to their mental health – attributing mental health symptoms to individual identity characteristics rather than the systems of oppression and power (e.g., sexism and ageism) behind them, or indeed circumstances leading to poor mental health. While older women regularly experience ageism, many participants felt that being older may potentially give them more power when it came to healthcare consultations about their mental health, as it would not be attributed to ‘hormones’ in the same way.

Participant 10’s testimony further reveals the intersectionality of her experiences of discrimination through interpersonal experiences of not being believed by individual practitioners. During the discussion, she mentioned her race ("*people don’t take you seriously sometimes with the colour of the skin*”), gender (“*females, we get seen as we complain a lot, we moan a lot, we just make a fuss out of something*”), age (“*you’re young, you’ll get over it*”) and language (“*you have to speak proper English… you are discriminated with your terminology*”), which reveals the connectedness of these systems of oppression, leading to a minimising of this participant’s mental health condition that was compounded by a layering of multiple marginalised positions.

Some participants reflected on how they felt they had to change their behaviour to try and navigate the discrimination embedded within the process of seeking mental health support. For instance, Participant 1 changed the way she communicated with healthcare professionals so she could be taken seriously through trying to appear older and less controlled by her emotions; resisting labels that had heretofore been placed on her as a young woman:

I learnt how to communicate as if I was older…I’ve had to learn how to be very unemotional when I’ve articulated anything about my mental health. Because, when I was younger and trying to have myself taken seriously, I think I was very upset…I’m quite cold and stony when talking about my mental health now, which people don’t question me so much on it [P1]

Several young women felt as though they had to “*prove*” they needed mental health treatment, therapy or support and that their symptoms were severe enough, again reflecting that being both young and a woman compounds experiences of symptom minimisation:

It got to a point where, in work, I felt like I had to cut myself to show that I’m in pain, because I never had proof to say that I’m going through something [P10]

...especially for myself I know from a mental health point of view if they can’t see it or if you can’t prove it with a doctor’s note that you’ve not been well, nobody takes you seriously [P22]

I don't think I ever went to the doctors at all, just because I felt a bit scared about going or just felt like what I would be going for wasn't reason enough. It wasn't justified. It's not real. [P21]

These excerpts reveal the internalisation of discriminatory discourses and interpersonal experiences. For some, this internalising made their mental health worse, due to “*confusion*” [P4] and “*self-doubt*” [P13] about their mental health symptoms. Others actively resisted internalising gendered labels through changing their behaviour, but recognised their privileged position in being able to do so (e.g., being highly educated, White or experienced with health professions/systems). This theme conveys that the lack of treatment for mental health problems experienced by young women is discriminatory and therefore a contributor to inequalities.

### Theme 3. The chain of dismissal

Participants exemplified how gendered mental health discourse permeates all aspects of their health, not just in accessing support for mental health conditions as explored in the previous theme. For instance, several participants told us about instances where they accessed healthcare due to physical health symptoms (e.g., UTIs, chronic fatigue, long covid, chronic pain, gynaecological issues, breathing problems, fainting), but they were told by healthcare professionals that it was probable that what they were experiencing was actually a mental health issue, namely anxiety. A few participants discussed the stereotypes around the trope of an “*anxious teenage girl*” [Participant 1], which in turn framed their physical symptoms as “*down to mental health*” more easily by healthcare professionals [Participant 11]:

I went in for my chronic issues, I went in, and it was always dismissiveness of it, always the talk of it being anxiety and depression [P16].

I used to struggle a lot with getting reoccurring UTIs as a teenager. It would always happen in periods when I was stressed or I’m down. But, because it was so interlinked with being an anxious teenage girl, it would almost be like a phantom UTI [P1]

He [the doctor] said anxiety and worrying all the time, but he’s mentioned that I probably have like [low] blood pressure… I’ve been like this for two years, but I don’t think it’s because it’s anxiety [P7]

Some of the participants accepted that it was entirely possible that their physical symptoms could be partially mental health related, but it was their experience of diagnosis being based on assumptions about them as a young women, often with “*no tests*” [Participant 11], “*no investigations*” [Participant 16] and usually few “*questions*” asked [Participant 15] about their symptoms. For example, this participant said:

The doctor presumed it was psychological, even though I had loads of physical symptoms and he never discussed it with me, at all. I felt like what he was saying was only to do with my age and me being a girl, it wasn’t because of any tests he’d done …I started doubting whether I was being dramatic about it and stuff, when I wasn’t [P11].

In instances where a mental health diagnosis or suggestion was given during these types of consultations for physical symptoms, this was not followed by the offer of mental health support. Young women interpreted the mental health diagnosis as a way of “*writing them off*” [P16] and getting them to go away and “*get on with it*” [P9] themselves, as opposed to actively pursuing a pathway of treatment for their symptoms. This experience resulted in young women feel like they had made up their physical pain or it was not “*real*” [P23] but also that any potential mental health issues were not seen as a serious health concern:

I’ve had the kind of ‘pain is in the brain’-type chat, which I think everybody who has chronic pain... We all know that. It doesn’t make it any less real and it doesn’t make it any easier to deal with it [P23].

It did also annoy me that they didn’t even want to help me, like if it was anxiety and depression like they said, they just wanted to write it down, and write me off, and they weren’t offering to help me [P16].

**Fig. 1:**
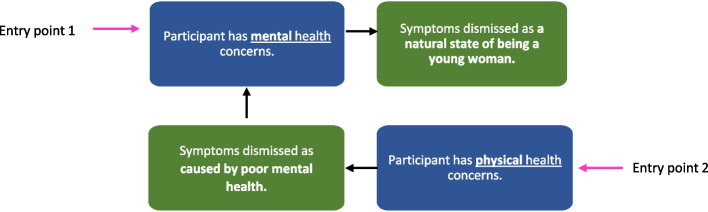
The Chain of Dismissal Legend: Entry Point 1 relates to participants seeking support in relation to their mental health. We find multiple instances across our sample of mental health symptoms being dismissed due to assumptions that they were synonymous with being young and female. Entry Point 2 relates to participants seeking healthcare support for physical health concerns, which were then attributed to psychological factors such as stress or anxiety. The reason this theory works as a chain is that, for some participants, the dismissal of symptoms and subsequent delays in support resulted in further health problems – either (1) the original health concern becoming worse, (2) mental health conditions developing or worsening as a result of stress in being dismissed

These experiences meant that some young women entered what we theorised as a ‘chain of dismissal’ (Fig. 1). For these women, regardless of whether they were seeking healthcare for mental or physical health symptoms, gender stereotypes connected to mental health were used as a way of dismissing them, and therefore fuelling health inequalities.

Entry point 1 was experienced by participants in the previous theme through their attempts to obtain mental health treatment. To provide some examples of how the chain played out through Entry Point 2, Participant 16 had physical health symptoms including extreme fatigue and breathing problems that were dismissed by healthcare professionals as anxiety. That experience of not being believed about her health condition meant she developed panic attacks, which were then explained by healthcare professionals as due to her gender and age. Treatment was either delayed or not provided at all for Participant 16’s physical or mental health symptoms. Further, Participant 21 shared her struggle in getting diagnosed with Attention deficit hyperactivity disorder (ADHD), in which she was not believed and told by healthcare professionals that it was probably a mental health issue, and how that experience “*bred anxiety and periods of depression*”:

I think the whole process of women just not being believed. And then, kind of, being chucked on antidepressants, especially when I would go to a man, health professional, I feel like they’d just be like, “Oh, yeah. Right.” It made me feel, like, you know, I was an irrational woman.

As a result of experiencing parts of the ‘chain of dismissal’, several young women reported not wanting to declare mental health symptoms during healthcare appointments as they suspected it would detract from the investigation of other health issues. Participants highlighted how the focus is always on that mental health “*record*” [P1] and therefore they are not listened to about other health concerns. For instance, Participant 1 claimed she was hesitant to “*be honest*” when it came to her mental health as it may “*automatically discredit*” her symptoms:

I was often worried that my [physical] health issues would not be investigated as the doctor could quite simply put it down to, “Oh, you’ve got a history of having some mental health issues.” P1]

Here, mental health status becomes an additional intersection driving inequalities in health. Participants acknowledged that mental health stigma is present for people of all genders and some noted male friends or family members who had suffered mental health issues and had felt less capable of asking for support than women. However, despite being comfortable talking about mental health and asking for support, our participants expressed a fear (based on real experiences) that a mental health diagnosis would act as a further barrier and compound the intersectional discrimination they experience within the health system.

## Discussion

This study explored the diverse and intersectional pathways through which discrimination shapes young women’s wellbeing and mental health. Our first key finding was that discrimination appears to amplify mental health problems for young women. Participants experiencing multiple marginalisation (including socioeconomic deprivation and racism) expressed a clear route between experiences of macro/meso level discrimination and their levels of stress, anxiety and depression, highlighting the distinctly intersectional nature of discrimination. There is a large body of work that supports this finding, most of which has focused on sexual and ethnic minority youth [39, 40]. What we contribute here is an illustration of how this experience is both intersectional and multi-level. Only aiming to reduce gender discrimination would obscure the reproduction of inequality and disadvantage [45]. Therefore, we underscore what other researchers have raised in that approaching gender equality in health policy in a simplistic and reductionist way, over and above other social inequalities, needs to be avoided and instead the nuance and diversity of the gendered experiences should be addressed [45]. More could be done across the UK to fully embed intersectionality into policymaking.

Anticipation of discrimination was also highlighted as a contributor to poor mental health. Constantly expecting to be treated unfairly and the practices young women put in place to navigate the discriminatory landscape created strain and exhaustion. Previous research has mostly examined this in relation to anticipated racism. For instance, Hope et al [46] highlight the anticipatory stress of future instances of racism for Black adolescents, which was stronger for those who had past experiences of racism at interpersonal, institutional and cultural levels. Young women in our sample contrast this idea to an extent in that the anticipation of racism was also felt by those who said they had not experienced an explicit incident of racism. Instead, it was often seeing family members experience discrimination and the broader cultural landscape of racism that heightened their expectation (an experience termed ‘vicarious racism’ [47]). Quinn et al [47] found that vicarious racism is common and may impact on health over and above direct discrimination, suggesting that our current understanding of the impact of discrimination is likely underestimated. Our finding supports this and illuminates that interventions and policies to try and reduce direct interpersonal acts of discrimination, through healthcare professional training for example, will do little to ease the burden marginalised women feel as they navigate certain spaces. These women expect discrimination regardless and that affects their mental health. Decolonising and dismantling the structures that support racism and other forms of unfair treatment is vital to begin to foster environments in which young women from marginalised groups can navigate freely without fear of discrimination.

Another key finding was that the lack of support for mental health was experienced as a form of discrimination. While young women qualified their experiences by recognising the pressures on services in the UK, they gave specific examples of how those pressures translated to an unequal experience of accessing mental health support. A common example was male peers at school being referred on or treated more quickly when raising mental health concerns, based on the assumption that women are more dramatic, their symptoms less serious and the causes of their mental health issues likely hormonal. This finding contributes to the literature around the framing of mental health issues, with girls often been viewed as having non-fatal, less visible and less urgent conditions [18]. Our finding also reflects research by Hamilton et al [48], which details how these structural level processes are conceptualised by individuals as discrimination and result in further marginalising certain groups. The authors found that macro discriminations resulted in delayed access to treatment, prolonged distress and poorer relationships with healthcare professionals [48]. Based on our findings, within the context of other evidence, we would recommend quantitative investigation, which is currently lacking, to map inequalities in mental health waiting times and referral times. This would help demonstrate the magnitude of the problem and develop strategies to make the mental health system more equitable.

Further, our finding that mental health status acts as an additional intersectional position that further marginalises young women chimes with a body of research on stigma and mental health conditions. For instance, Rai et al [27] draw on insights from people from disadvantaged backgrounds with stigmatized health conditions. They highlight how the intersectional experience of stigma compounded with other inequalities such as ‘gender, sexuality and poverty can lead to social exclusion, isolation, hamper access to health services, employment and education’ ([27], p.2). In recognition of the impact of stigma, The Lancet Commission on ending stigma and discrimination in mental health was launched [49]. The Commission highlights that mental health stigma can be viewed within the context of other types of discrimination using an intersectional lens, but there are special characteristics of mental health stigma. For example, mental health conditions are linked to self-responsibility and factors within a person’s control, which separates them in part from other health conditions [49]. We would argue that our findings contribute to this understanding. Young women in our sample felt that healthcare professionals found it “*easier*” to determine their symptoms were: (i) related to mental health and (ii) strongly tied to their age and gender, rather than investigating the possibility of a physical health condition. This process framed young women’s health as their own responsibility. We could infer that this embedded neoliberal discourse within the treatment of mental health results in a unique position of vulnerability for young women with mental health conditions. Their positionality is reinforced by mental health stigma, which makes it extremely difficult to be taken seriously and ultimately access healthcare.

Finally, we generated a social theoretical model from the data that we termed the ‘chain of dismissal’. This model illustrates how gender stereotypes connected to mental health are embedded within health systems and professionals and create inequalities in access to healthcare for *both* mental and physical health. Research informing the Women’s Health Strategy for England [50] highlighted similar cultural discourse on gender and mental health. Their survey ‘Women’s Health – Let’s talk about it’ [51] generated 100,000 responses from women in England and suggested that women’s symptoms (either mental or physical) were regularly framed as synonymous with being a woman due to hormones and menstruation [51]. Our findings convey that women are trying to be taken seriously and get proper support for their mental health, while many also want their physical health conditions to be properly investigated and not instantly classified as a mental health problem. Young women find it impossible to achieve these things simultaneously (if they have both physical and mental health problems), with many resorting to masking or withholding their mental health status in the hopes their physical health condition will stand a chance of being investigated. We therefore underscore mental health inequalities as a social justice issue [27] and equality as a public health priority [52]. It is only through seeking to improve the cultural landscape and deconstructing damaging stereotypes about women and mental health, that we will begin to see improvements in young women’s health and reductions in health inequalities.

### Limitations

The current study presents some limitations. There were undoubtedly some women aged 16-25 who did not participate, who would have added a different perspective to the research. For example, mothers under 25 and young women from ethnic communities such as Gypsy, Roma and Traveller communities were missing from our study, despite being in unique intersectional positions that may have been important for experiences of discrimination and health in Scotland. We also only interviewed one gender non-conforming person, which may have been as a result of the recruitment and advertising methods. Nevertheless, the findings contain a range of experiences and perspectives that speak to several, regularly marginalised positions. Specifically, the sample had relatively high ethnic diversity and each quintile on the Scottish Index of Multiple Deprivation was represented. We highlight that many of our examples from the data were based on racism, but we must acknowledge that these experiences related to fewer than half of sample participants. As every participant from an ethnic minority background framed many of their experiences in relation to racism, we felt it was critical to explore this type of discrimination thoroughly. This qualitative study set out to understand how discrimination may affect women’s health in an intersectional way by exploring young women’s perspectives and experiences. They are therefore not generalisable to the population of Scottish women or individuals who can use women’s health support. The findings are also not representative of everyone experiencing mental health problems in Scotland. A further challenge relates to how intersectionality was employed in our study. Intersectionality seeks to understand various identity characteristics as equally relevant and largely researchers should try not to position one experience of discrimination as the most important. Yet, because this project had a focus on young women, we unavoidably privileged gender, sex and age and these were regularly the focus of participants’ experiences.

### Implications for policy

The findings from this work are already stimulating discussions with policymakers in Scotland, having been presented to individuals working in different areas of healthy policy (e.g., women’s health, mental health, primary care). The intersectional approach to data collection and analysis has allowed us to elucidate the experiences of women at varying positions of disadvantage. Our findings emphasize that health and healthcare policy intended to improve women’s health should strongly consider different intersections; a ‘one size fits all’ policy is unlikely to be equitable [53]. To begin to narrow inequalities and increase access to healthcare and mental health treatment for women, services and policies should be tailored to specific needs of minority groups. There needs to be stronger consideration of how young age compounds the experience of mental health discrimination that women experience. Further, there should be greater appreciation of gender, through gendering health policy and programme design [1], across health inequalities research and policy that in the UK currently focuses on socioeconomic circumstances [54]. Embedding this intersectional approach within research and policy presents a promising way forward to achieving health equity.

## Conclusions

The current study contributes to growing evidence on intersectional discrimination and its relationship with mental health. We found that young women experience intersectional and multi-level discrimination, which translates into stress and anxiety, particularly for those at positions of multiple disadvantage. The process of accessing mental health treatment was experienced by many as in itself discriminatory, with stereotypes around the gender/age intersection primarily driving unequal access. Young women highlighted that their age, gender, mental health status and ethnicity were salient parts of their ‘self’ that would result in low credibility and low legitimacy of any symptoms they would report. The impact of this intersectional discrimination was far-reaching beyond mental health, and limited young women’s access to other healthcare due to repeated dismissal by healthcare professionals. To improve mental health and reduce health inequalities for young women, multi-level approaches are needed, with strong consideration of how the structural and cultural landscape as well as individual healthcare professionals have critical implications for young women’s health. Overall, this study illuminates intersectional gender equality as a public health priority and mental healthcare access as a social justice issue.

### Supplementary Information

**Supplementary Material 1.**

## Data Availability

The qualitative dataset in this study is not available under the conditions by which the study was granted ethical approval by the Scottish Government, due to the sensitive nature of the content and difficulty anonymising the data.
