# Supplementary material for: Intersectional discrimination and mental health inequalities: a qualitative study of young women’s experiences in Scotland
Source: Int J Equity Health. 2024 Feb 29;23:45. doi: 10.1186/s12939-024-02133-3 (PMC10903064; doi:10.1186/s12939-024-02133-3)
Supplement: Supplementary file 1 — Supplementary Material 1. [file 12939_2024_2133_MOESM1_ESM.docx]

**INTERVIEW TOPIC GUIDE**

**Discrimination and Health – Women’s Health Plan**

This interview is on discrimination and health. This research is being carried out as part of the Scottish Government's Women's Health Plan, in which the plan and its associated work is inclusive of women, girls, and all those who access women's health services. We are interested in any and all experiences you are comfortable sharing on this topic. There are no right or wrong answers and if you don’t understand anything or we aren’t clear, do say, as you probably aren’t the only one thinking it.

Let’s get started then. We’ll begin with some questions about the terms used.

Definitions questions

1. What comes to mind when you think about your health and being ‘healthy’?
2. What things do you think impact on your health?
3. One of the things that potentially impacts on your health is discrimination. What does the word ‘discrimination’ mean to you?

One definition of discrimination is we found is: the unfair treatment of people based on certain characteristics including their sex, gender, race/ethnicity, disability, age, religion, education level, where you live, language and sexual orientation. This might be something experienced through an interaction with a person, an institution (like a workplace, school/college or the NHS) or a public space or environment. So we aren’t just talking about obvious instances of discrimination, they might be more subtle or hidden, and someone may experience several forms of discrimination at one time. This doesn’t mean *all* the negative experiences you have had, but the things that have happened to you specifically because of your background or belonging to a certain group.

1. Based on what you’ve told me and this definition, can tell me about a particular event when you experienced or witnessed discrimination? I won’t interrupt, go into as much detail as you like.

- *Probing question*: do you think that experience impacted on yours/the person’s health in any way?
- *Probing question*: do you have any reflections on why you think that was happening?
- Probing question: can you think of any other examples in/out of a healthcare setting? In an education/work setting? (*depending on which area they have focused on*)

1. Do you think any of these situations of discrimination you’ve described relate to specific parts of your identity (e.g. gender, race, age, sexual orientation, social class or anything else)?

- *Probing*: Why/why not? What do you think is happening there?
- *Probing*: In the situation you mentioned, is there one identity that is more important than the others? For example, do you think it’s because of your [e.g. gender] more so, your [e.g. race] more so, or is it both?

1. Thinking about where you live, can you tell me what kinds of things you think make it easier or harder to be ‘healthy’?

- Finances
- Options for activities (e.g. different kinds of sports)
- Services (health, education, transport)
- Local environment (green space, air quality)
- Behaviours (activity, diet)
- People (friends, peers, family, community, online influencers etc.)

1. Do you think there is discrimination (e.g. some people are unfairly excluded) happening in your example? How/how not?
2. *If there is time*: Before the interview, had you thought that discrimination could impact on your health?

**General probing questions throughout:**

- Why do you think that is?
- What do you think might be happening there?
